# Liver Transplant Outcomes in Patients With Transjugular Intrahepatic Portosystemic Shunt: A Retrospective Cohort Study With Propensity Score Analysis

**DOI:** 10.1016/j.jceh.2026.103561

**Published:** 2026-05-15

**Authors:** Marco Biolato, Quirino Lai, Tiziano Galasso, Giuseppe Marrone, Luca Miele, Antonio Liguori, Annamaria Amodeo, Lucia Monastero, Andrea Contegiacomo, Roberto Iezzi, Gabriele Spoletini, Salvatore Agnes, Antonio Grieco, Antonio Gasbarrini, Maurizio Pompili, Alfonso Avolio

**Affiliations:** ∗ Department of Medical and Surgical Sciences, Fondazione Policlinico Universitario Agostino Gemelli IRCCS, Rome, Italy; † Department of Translational Medicine and Surgery, Catholic University of Sacred Heart, Rome, Italy; ‡ Department of General and Specialty Surgery, Azienda Ospedaliero-Universitaria Policlinico Umberto I, Rome, Italy; § General Surgery and Organ Transplantation Unit, Sapienza University of Rome, Italy

**Keywords:** portal hypertension, postoperative complications, stent migration, ascites, variceal bleeding

## Abstract

**Background:**

Transjugular intrahepatic portosystemic shunt (TIPS) is used to manage portal hypertension–related complications in patients awaiting liver transplantation. However, the role of pretransplant TIPS placement remains controversial.

**Methods:**

We conducted a retrospective, single-center cohort study to evaluate the impact of pretransplant TIPS on postoperative complications and in-hospital mortality.

**Results:**

We included 263 cirrhotic patients who underwent liver transplantation from deceased donors between 2015 and 2023. Twenty-three patients (8.7%) underwent pretransplant TIPS placement. All TIPS were placed using controlled-expansion stents, with a median diameter of 8 ± 1 mm. Stent migration was identified intraoperatively in 7 patients (30.4%) and was associated with increased surgical complexity. After propensity score weighting, pretransplant TIPS was not independently associated with operative time, use of venovenous bypass, transfusion requirements, length of intensive care unit (ICU) or hospital stay, comprehensive complication index, major complications (Clavien–Dindo ≥ III), or in-hospital mortality. Conversely, reoperation rates were higher in the TIPS group (17.4% vs. 8.0%, *P* = 0.003). In multivariable analysis, previous abdominal surgery was the only independent predictor of in-hospital mortality.

**Conclusions:**

Pretransplant TIPS may allow patients with severe portal hypertension to achieve postoperative outcomes comparable to those without TIPS, but should be reserved for guideline-supported indications, given the lack of demonstrated survival benefit.

## Graphical abstract

**Figure:**
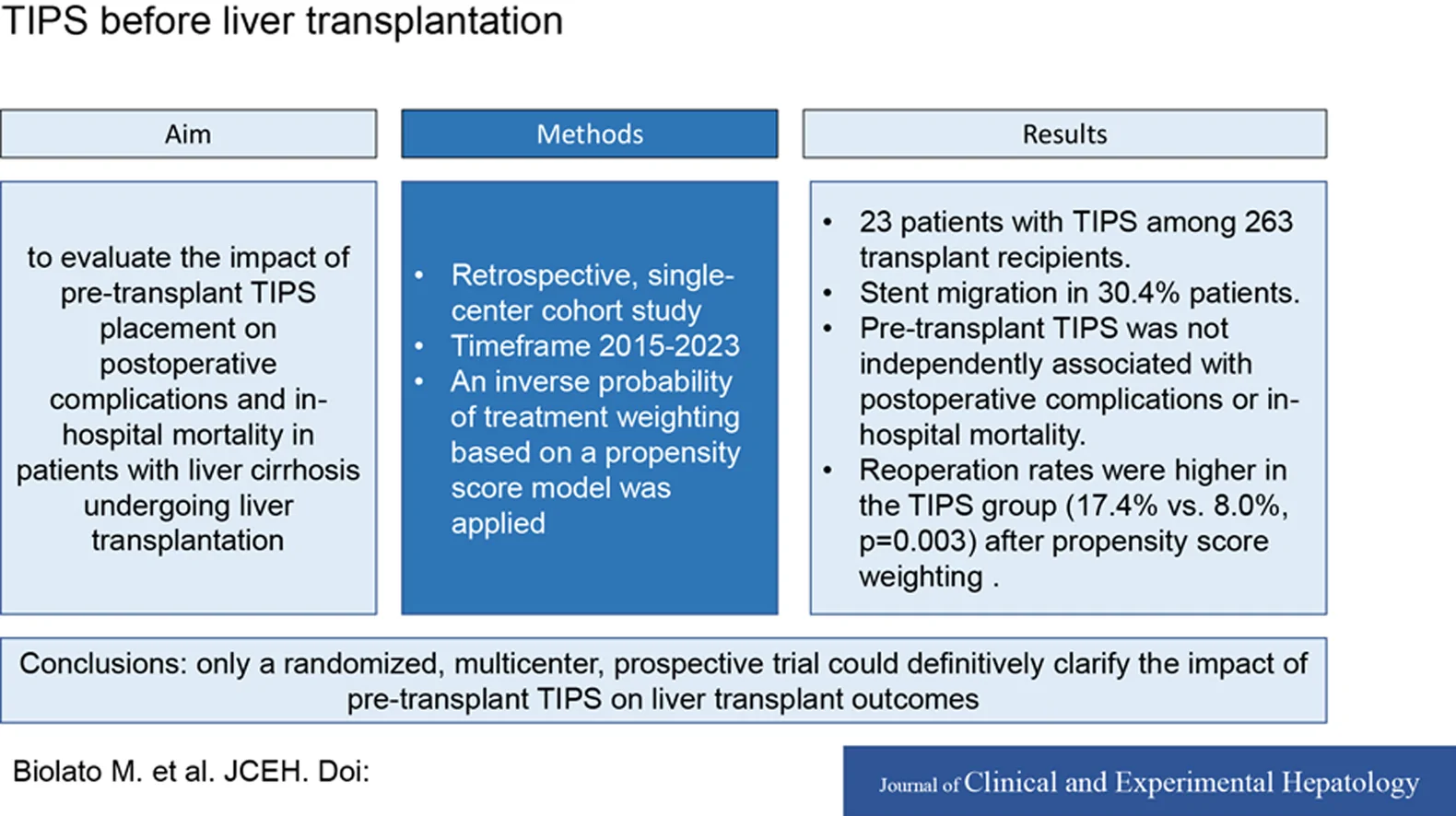

Liver transplantation (LT) is the standard of care for patients with life-threatening liver disease.^1^ Placement of a transjugular intrahepatic portosystemic shunt (TIPS) in a LT candidate presents many challenging aspects.^2^^,^^3^ Key strengths include treatment of portal hypertension–related bleeding^4^ and refractory or recurrent ascites,^5^ management of portal vein thrombosis (PVT) when anticoagulant therapy is ineffective or not tolerated, or in combination with percutaneous portal vein recanalization (PVR-TIPS) in patients with portal cavernoma,^6^^,^^7^ expected increase in waitlist survival,^8^ and potential improvement in nutritional status.^9^ On the other hand, TIPS placement carries risks such as misplacement or migration, which can increase the complexity of the transplant procedure;^10^ shunt-related complications, such as the development or worsening of hepatic encephalopathy (HE), cardiac decompensation, or deterioration of residual liver function; and the risk of paradoxical prolongation of waitlist time due to model for end-stage liver disease (MELD) score improvement.^2^^,^^8^^,^^11^

Recently, the role of TIPS as a strategy to reduce surgical risk in cirrhotic patients undergoing surgery has been increasingly recognized.^12^ In particular, Piecha *et al.* published a retrospective cohort study including 64 cirrhotic patients who underwent TIPS (primarily for ascites) within three months of nontransplant surgery, demonstrating a significant reduction in in-hospital mortality (19% vs. 40%) and postoperative complications.^13^ However, evidence regarding the effects of pretransplant TIPS on perioperative outcomes and in-hospital mortality in LT recipients remains limited and controversial. The aim of the study is to evaluate the impact of pretransplant TIPS placement on postoperative complications and in-hospital mortality in patients with liver cirrhosis undergoing LT.

## PATIENTS AND METHODS

### Study Design

This was a retrospective, single-center, observational cohort study. All patients with liver cirrhosis who underwent liver transplantation from deceased donors between 2015 and 2023 at the Fondazione Policlinico Universitario Agostino Gemelli IRCCS in Rome were included. Patients were excluded if they had undergone liver retransplantation, combined liver–kidney transplantation, or transplantation for indications other than liver cirrhosis (e.g. acute liver failure, polycystic liver disease, Caroli disease, Rendu–Osler disease, Budd–Chiari syndrome, acute post-traumatic liver injury, or unresectable hilar cholangiocarcinoma). Cirrhosis was defined based on a combination of clinical, laboratory, imaging, and elastographic criteria.^14^ The study protocol was approved by the Territorial Ethics Committee Lazio Area 3, IRCCS Policlinico Gemelli.

### TIPS Placement Procedure

The decision to place the TIPS was made after a multidisciplinary discussion involving hepatologists, transplant surgeons, and interventional radiologists. In selected patients with a history of controlled or recurrent hepatic encephalopathy, TIPS placement was considered when the expected benefits (e.g. management of refractory ascites or variceal bleeding) were deemed to outweigh the potential risks, based on multidisciplinary evaluation. Whenever feasible, 8-mm-diameter stents were used, as they have been associated with a lower risk of post-TIPS hepatic encephalopathy compared to 10-mm stents.^15^^,^^16^ All patients with a history of hepatic encephalopathy prior to TIPS continued treatment with rifaximin and lactulose according to current guideline recommendations.^4^ All TIPS procedures were performed on an inpatient basis under general anesthesia or deep sedation in an angiography suite equipped with a Philips Azurion M20 system. All interventions were conducted by an experienced team of interventional radiologists in accordance with the Cardiovascular and Interventional Radiological Society of Europe (CIRSE) Standards of Practice Guidelines.^17^

Vascular access was obtained via the right internal jugular vein under ultrasound guidance, followed by catheterization of the right or middle hepatic vein using a hydrophilic guidewire and a multipurpose diagnostic catheter. The right main portal vein was then punctured under real-time transabdominal ultrasound guidance using either a Rösch–Uchida or Colapinto needle set. The guidewire was advanced into the portal vein lumen, and a catheter was subsequently introduced. Diagnostic portography was performed to confirm correct catheter placement, assess portal venous anatomy, and determine the intrahepatic parenchymal tract length.

The intrahepatic tract was dilated using a 6 mm or 8 mm angioplasty balloon (Mustang, Boston Scientific), followed by deployment of a controlled-expansion polytetrafluoroethylene (PTFE)-covered stent graft (Viatorr, Gore) across the portosystemic tract. The portosystemic pressure gradient (PSG)—defined as the difference between portal vein pressure and inferior vena cava pressure—was measured before and after stent deployment. If the hemodynamic target (PSG <12 mmHg or ≥40% reduction from baseline) was not achieved, additional balloon dilatation was performed, up to a maximum diameter of 10 mm.

Postprocedure follow-up included clinical, biochemical, and Doppler ultrasound assessments.

### Study Outcomes

The primary outcome was in-hospital mortality. Secondary outcomes included postoperative complications occurring during the index hospitalization for liver transplantation, rate of reoperation, length of ICU stay, and total hospital stay. Intraoperative variables were also analyzed, including operative time, transfusion requirements for blood products (packed red blood cells, platelet pools, and plasma units), and intraoperative evidence of TIPS migration.

### Data Collection and Variables

Electronic medical records were retrospectively reviewed. Collected variables included demographic data (age at transplantation, sex, blood type, and body mass index [BMI]), indication for LT (liver cirrhosis related to hepatitis C virus [HCV], hepatitis B virus [HBV], alcohol-related liver disease, metabolic-associated steatotic liver disease [MASLD] or other rare causes, as well as the presence of hepatocellular carcinoma [HCC]), PVT on pretransplant imaging, comorbidities including chronic obstructive pulmonary disease (COPD), chronic kidney disease (stages III–V), arterial hypertension, diabetes mellitus, ischemic heart disease (defined as previous acute myocardial infarction or chronic ischemic disease requiring revascularization), and a history of previous supramesocolic surgery (excluding laparoscopic cholecystectomy).

The MELD^18^ and MELD-sodium (MELDNa) scores^19^ were documented at multiple time points: at listing (with MELDNa being the reference score in our region), at TIPS placement, 4 weeks after TIPS placement, and at transplantation. Details of portal hypertension-related complications were assessed, including history of esophageal varices, congestive gastropathy, hepatic encephalopathy, ascites, and hepatorenal syndrome.

TIPS-specific variables included stent diameter (mm), incidence and grade of post-TIPS hepatic encephalopathy according to the West Haven criteria,^20^ interval between TIPS placement and transplantation, and biochemical parameters measured before and 4 weeks after TIPS placement (serum ammonia, total bilirubin, albumin, sodium, creatinine, hemoglobin, and INR). Diuretic therapy (furosemide and aldosterone antagonists) was also recorded at the same time points.

Intraoperative variables, collected from the operative report, included donor age, intraoperative PVT, TIPS dislocation at the time of transplantation, operative time (minutes), use of venovenous bypass during the anhepatic phase, and transfusion requirements (number of packed red blood cells, platelet pools, and plasma units).

Postoperative data included length of ICU stay, total hospital stay, in-hospital mortality, reoperation rate, and postoperative complications during the index hospitalization. Complications were graded according to the Clavien–Dindo classification^21^ and summarized using the comprehensive complication index (CCI^22^).

### Statistical Analysis

Baseline donor, recipient, and transplant-related characteristics were compared between patients with and without TIPS. Continuous variables were reported as median (interquartile range [IQR]) and compared using the Mann–Whitney U test, while categorical variables were presented as counts and percentages and compared using the Chi-square or Fisher's exact test, as appropriate.

To adjust for baseline differences, inverse probability of treatment weighting (IPTW) based on propensity scores was applied. Propensity scores were estimated using a logistic regression model including only pretransplant covariates, with the most predictive variables selected via LASSO (Least Absolute Shrinkage and Selection Operator). IPTW weights created a pseudo-population with balanced covariates, assessed through standardized mean differences (Cohen's d) before and after weighting.

Postoperative outcomes in the weighted pseudo-population were compared using the same tests, weighted by IPTW. A multivariable logistic regression model for in-hospital mortality was then constructed using IPTW-weighted maximum likelihood estimation. A parsimonious model was derived by retaining the TIPS variable regardless of its statistical significance and selecting no more than two additional covariates identified by LASSO. Beta-coefficients, standard errors (SE), odds ratios (OR), 95% confidence intervals (CI), and *P*-values were reported. The *P*-values <0.05 were considered statistically significant.

## RESULTS

**Figure 1:**
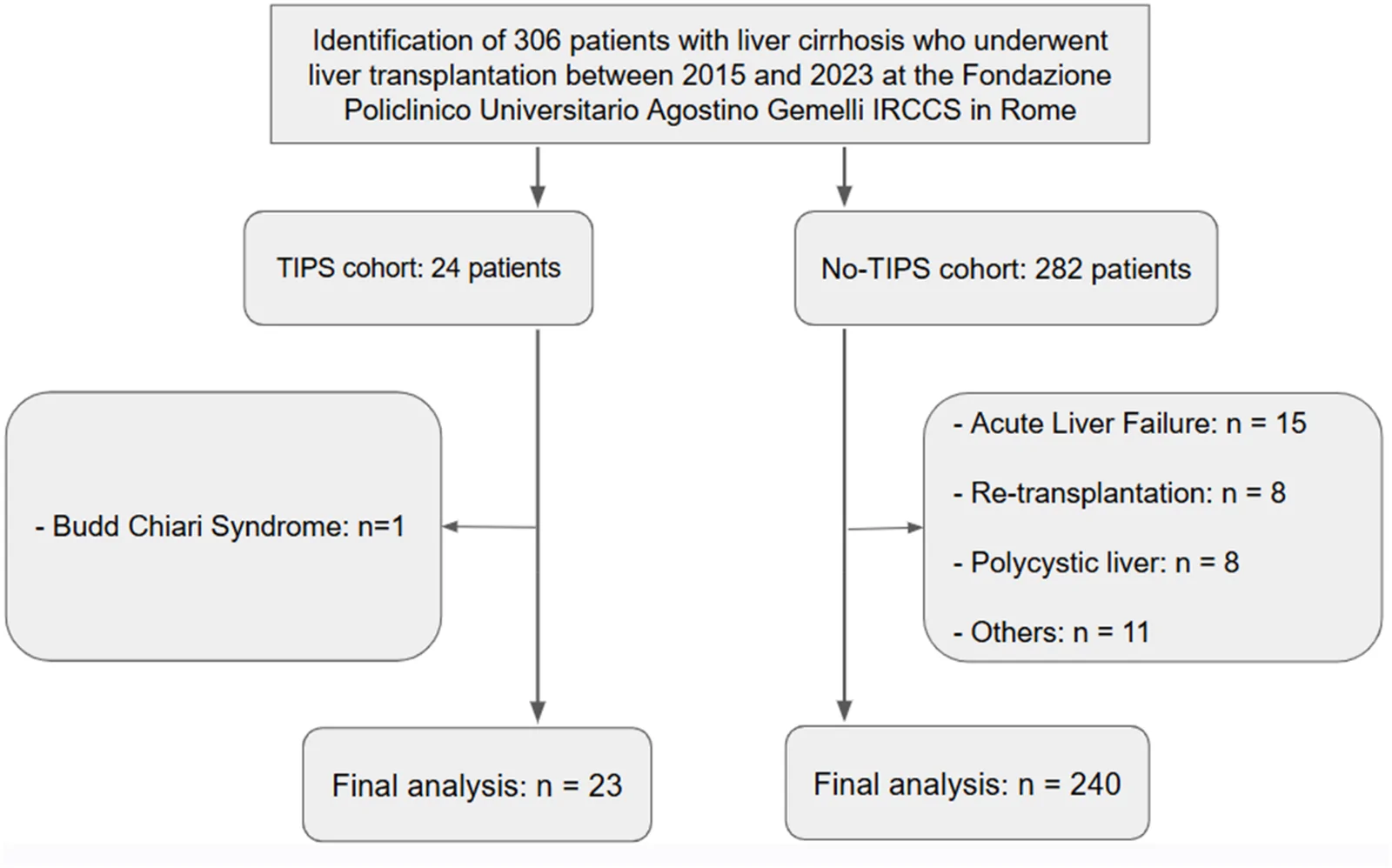
Study population flow diagram.

A total of 306 patients underwent LT at Fondazione Policlinico Universitario Agostino Gemelli IRCCS, Rome, between 2015 and 2023. Patients were divided into two groups: the TIPS group and the No-TIPS group. The TIPS group comprised 24 patients; one was excluded from analysis due to a diagnosis of Budd–Chiari syndrome. The No-TIPS group included 282 patients. Of these, 42 were excluded for the following reasons: acute liver failure (n = 15), liver retransplantation (n = 8), polycystic liver disease (n = 8), and other conditions (n = 11). Ultimately, 23 patients were included in the TIPS group and 240 patients in the No-TIPS group for the final analysis (Figure 1). Over the same period (2015–2023), our center managed 44 LT candidates who underwent TIPS placement. Of these, 16 were waitlisted; among them, 5 were subsequently delisted due to clinical improvement, and 11 were removed from the waiting list due to worsening clinical conditions. Among the 28 candidates who were not listed for LT, 8 did not meet the MELD score criteria for listing, 18 had contraindications to LT, and 2 died from complications related to TIPS placement.

### TIPS Population

**Figure 2:**
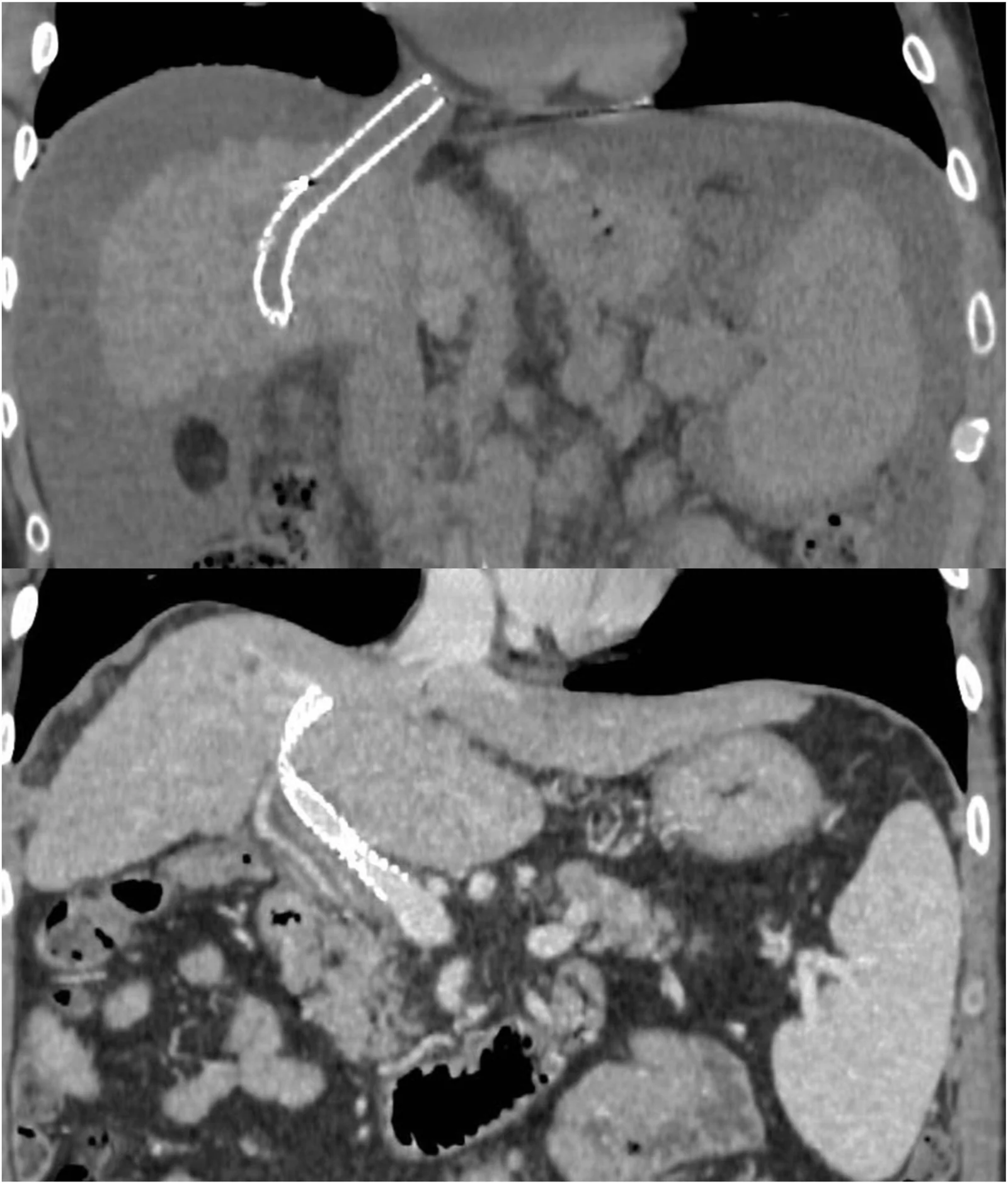
Two examples of stent migration on pre–liver transplant imaging.

Characteristics of the 23 patients undergoing TIPS are summarized in Table 1. The indications for TIPS placement were refractory or recurrent ascites (n = 15, 65.2%), and portal hypertension-related bleeding (n = 8, 34.8%). A controlled-expansion stent was used in all patients, with a median diameter of 8 ± 1 mm. The procedure was performed at our center in 17 patients (73.9%). Postprocedural portosystemic encephalopathy occurred in 12 patients (52.2%), with the following severity according to the West–Haven classification: grade I in 3 patients (25%), grade II in 6 (50%), grade III in 2 (16.7%), and grade IV in 1 (8.3%). None of the patients required a reduction in stent diameter due to refractory hepatic encephalopathy. The onset of post-TIPS encephalopathy did not preclude successful LT in any case. The median interval between TIPS placement and LT was 8 months (IQR 3–24.5). Stent migration was identified intraoperatively in 7 patients (30.4%), occurring toward the portal side in 4 patients, toward the caval side in 1 patient, and involving both sides in 2 patients (Figure 2). Intraoperative management of stent migration involved a temporary portocaval anastomosis in 3 cases, stent extraction from the portal end in 2 cases, and from the caval end in 1 case, which was complicated by a minor vascular wall tear. Construction of a venous jump graft was not required in any case. The median interval between TIPS placement and LT did not differ significantly between the seven patients with TIPS migration (11 months, IQR: 5.5–22.5) and the sixteen patients without migration (8 months, IQR: 2.75–26.5; *P* = 0.67).

**Table 1:** Characteristics of 23 Patients Undergoing TIPS.

Indication to TIPS	
- Refractory or recurrent ascites	15 (65.2%)
- Prevention of variceal bleeding	8 (34.8%)
Controlled-expansion stents	16 (100%)^a^
Stent caliber (mm),	8 ± 1
Positioned in Gemelli hospital	17 (73.9%)
HE post-TIPS	12 (52.2%)
HE grade post-TIPS	
- Grade I	3 (25%)
- Grade II	6 (50%)
- Grade III	2 (16.6%)
- Grade IV	1 (8.4%)
Interval between TIPS placement and OLT (months)	8 ± 22
TIPS migrated at transplant	7 (30.4%)

Values are expressed as Median [IQR] or n (%).

TIPS = transjugular intrahepatic porto-systemic shunt; HE = hepatic encephalopathy.

a Missed data for seven patients.

Comparison of clinical and laboratory parameters before and 4 weeks after TIPS placement revealed a significant increase in serum bilirubin levels (from 2.0 ± 1.0 to 3.0 ± 2.0 mg/dL; *P* = 0.009), along with a significant reduction in the daily dose of antialdosterone agents (from 200 ± 188 to 100 ± 113 mg/day; *P* = 0.038). No statistically significant changes were observed in INR, creatinine, sodium, albumin, hemoglobin, or ammonia levels, nor in MELD and MELDNa scores or furosemide dosage (Supplementary Table).

### Univariate Analysis

Among the 263 patients included in the study cohort, 225 (85.6%) were male, with a median age at listing of 58 years. The most frequent etiologies of liver disease were alcohol-related liver disease (48.3%), MASLD (28.1%), HBV infection (27.0%), and HCV infection (23.9%). HCC was present in 52.9% of patients. The median BMI was 26.2. The most common comorbidities included diabetes mellitus (40.3%), arterial hypertension (32.3%), COPD (11.0%), and ischemic heart disease (10.3%). A history of previous supra-mesocolic surgery was documented in 15.2% of patients. The median MELDNa score was 15 both at listing and at transplantation. No significant differences were observed between the TIPS and no-TIPS groups in baseline characteristics (Table 2).

**Table 2:** Donor-, Recipient-, and Transplant-related Characteristics Before IPTW.

Variable	Whole cohort (n = 263)	TIPS (n = 23)	No TIPS (n = 240)	*P*-value
Male sex	225 (85.6%)	19 (82.6%)	206 (85.8%)	ns
Age at listing	58 [52–63]	60 [52–63]	58 [52–63]	ns
HCC	139 (52.9%)	8 (34.8%)	131 (54.6%)	ns
HCV	61 (23.9%)	3 (13.0%)	60 (26.2%)	ns
HBV	71 (27.0%)	9 (39.1%)	62 (25.8%)	ns
Alcohol-related liver disease	127 (48.3%)	11 (47.8%)	116 (48.3%)	ns
MASLD	74 (28.1%)	8 (34.8%)	66 (27.5%)	ns
Other etiologies	24 (9.1%)	1 (4.3%)	23 (9.6%)	ns
BMI (kg/m^2^)	26.2 [23.8–29.6]	26.4 [23.8–29.5]	26.2 [23.8–29.6]	ns
Portal vein thrombosis at listing	35 (13.3%)	2 (8.7%)	33 (13.7%)	ns
COPD	29 (11.0%)	5 (21.7%)	24 (10.0%)	ns
Chronic kidney disease stage III–V	11 (4.2%)	1 (4.3%)	10 (4.2%)	ns
Ischemic heart disease	27 (10.3%)	3 (13.0%)	24 (10.0%)	ns
Diabetes	106 (40.3%)	11 (47.8%)	95 (39.6%)	ns
Arterial hypertension	85 (32.3%)	6 (26.1%)	79 (32.9%)	ns
Previous supra-mesocolic surgery^b^	40 (15.2%)	2 (8.7%)	38 (15.8%)	ns
Esophageal/gastric varices	177 (67.3%)	22 (95.6%)^a^	155 (64.5%)	0.001
History of variceal bleeding	42 (16.0%)	11 (47.8%)^a^	31 (12.9%)	0.000
History of hepatorenal syndrome	18 (6.8%)	4 (17.4%)^a^	14 (5.8%)	ns
Ascites	181 (68.8%)	22 (95.6%)^a^	159 (66.2%)	0.004
Hepatic encephalopathy	115 (43.7%)	11 (47.8%)^a^	104 (43.3%)	ns
Portal vein thrombosis at LT	30 (11.4%)	2 (8.7%)	28 (11.6%)	0.017
MELDNa at listing	15 [12–21]	15 [12–20]	16 [12–20.5]	ns
MELDNa at transplant	15 [12–25]	18 [12–25]	15 [12–25]	ns
Donor age	63 [49–74]	63 [48–71]	63 [52–73]	ns
Use of venovenous bypass	41 (15.6%)	3 (13.0%)	38 (15.8%)	ns
Surgery duration (minutes)	642.0 [568–728.5]	670.0 [574.5–704.0]	642.0 [569.5–729.2]	ns
Units of red blood cell transfused	4.0 [1.0–7.0]	6.0 [3.0–7.5]	4.0 [1.0–7.0]	ns
Units of platelets transfused	0.0 [0.0–1.0]	0.0 [0.0–1.0]	0.0 [0.0–1.0]	ns
Units of plasma transfused	0.0 [0.0–5.0]	3.0 [0.0–5.0]	0.0 [0.0–5.0]	ns
ICU stay after transplant	5.0 [3.0–9.0]	6.0 [3.0–8.0]	5.0 [3.0–9.0]	ns
Total post-transplant hospital stay	21.0 [15.0–31.25]	20.0 [14–23.0]	21.0 [15.0–32.5]	ns
CCI	34.6 [22.6–52.4]	39.5 [22.6–54.0]	34.6 [22.6–52.4]	ns
Clavien–Dindo ≥ grade IIIb	76 (29.1%)	8 (36.3%)	68 (28.4%)	ns
Reoperation	34 (12.9%)	5 (21.7%)	29 (12.1%)	ns
Intraoperative mortality during LT	4 (1.5%)	1 (4.3%)	3 (1.2%)	ns
In-hospital mortality after LT	19 (7.2%)	2 (8.7%)	17 (7.1%)	ns

Values are expressed as median [IQR] or n (%).

Mann–Whitney U test or Chi-square test.

IPTW = inverse probability of treatment weighting; TIPS = transjugular intrahepatic porto-systemic shunt; HCC = hepatocellular carcinoma; HCV = hepatitis C virus; HBV = hepatitis B virus; MASLD = metabolic-associated steatotic liver disease; BMI = body mass index; COPD = chronic obstructive pulmonary disease; LT = liver transplantation; MELD = model for end-stage liver disease; MELDNa = MELD-sodium; ICU = intensive care unit; CCI = comprehensive complication index.

a Before TIPS placement.

b Excluding laparoscopic cholecystectomy.

A total of 30 patients (11.4%) had an intraoperative finding of PVT involving the main portal trunk, including 2 patients (8.7%) in the TIPS group and 28 patients (11.6%) in the no-TIPS group (*P* = 0.017, Table 2). In the TIPS group, one case represented an intraoperative diagnosis in a patient with a previously patent portal venous system on preoperative imaging, while the second patient had a known PVT and was receiving anticoagulation with enoxaparin at the time of transplantation. PVR-TIPS was not undertaken in this patient. In the no-TIPS group, 5 patients had an intraoperative diagnosis of PVT. Nineteen patients were receiving anticoagulant therapy (14 with enoxaparin or fondaparinux and 5 with warfarin); however, in 3 cases, anticoagulation had been discontinued before transplantation due to bleeding complications. The remaining 4 patients did not receive anticoagulation because of severe thrombocytopenia and/or advanced liver dysfunction.

Patients who underwent TIPS had a higher prevalence of a history of portal hypertension–related complications (Table 2). Specifically, esophageal or gastric varices were more common in the TIPS group than in the no-TIPS group (95.6% vs. 64.5%, *P* = 0.001), as were a history of variceal bleeding (47.8% vs. 12.9%, *P* < 0.001) and ascites (95.6% vs. 66.2%, *P* = 0.004). Hepatic encephalopathy was common in both groups (47.8% vs. 43.3%), with no significant difference (*P* = 0.93).

Perioperative outcomes did not differ significantly between the two groups (Table 2). In the whole population, the median donor age was 63 years, median operative time 642 min, median packed red blood cell units transfused 4, median platelet and plasma units (200 mL) transfused 0. The median length of ICU stay was 5 days, and total hospital stay was 21 days. The median CCI was 34.5. Major complications (Clavien–Dindo grade ≥ III) occurred in 29.1% of patients. The reoperation rate was 12.9%, with 5 reoperations in the TIPS group (21.7%) and 29 in the no-TIPS group (12.1%), with no significant difference observed (*P* = 0.19). In the TIPS group, 4 reoperations were for biliary complications and 1 for bleeding. Patients with TIPS migration at the time of transplantation exhibited a higher reoperation rate (42.9% vs. 12.5%), although this difference did not reach statistical significance (*P* = 0.10). Finally, the intraoperative mortality was 1.5%, and in-hospital mortality was 7.2%. A total of 19 in-hospital deaths occurred in our cohort. In the TIPS group, two patients died: one due to intraoperative cardiac arrest and one due to sepsis. In the no-TIPS group, mortality was predominantly related to surgical or technical complications (n = 9), followed by infectious complications (n = 6); additionally, one patient died from intraoperative cardiac arrest and one from acute respiratory distress syndrome (ARDS).

### Balance Assessment Before and After Weighting

Covariate balance between the TIPS and control groups was assessed using standardized mean differences (Cohen's d). Prior to weighting, several baseline characteristics exhibited moderate to large imbalances, notably ascites (d = −0.64), COPD (d = −0.44), HBV infection (d = −0.33), and HCC (d = 0.40). Following IPTW, the majority of covariates achieved satisfactory balance, although residual imbalances remained for ascites (d = −0.48) and HCC (d = 0.36), both within a tolerable range for weighted comparisons. Overall, IPTW provided a pseudo-population sufficiently balanced for subsequent outcome analyses (Table 3).

**Table 3:** Comparison of Baseline Clinical Characteristics Between TIPS and No-TIPS Groups Before and After IPTW Adjustment.

Variable	Pre-IPTW			Post-IPTW		
	No TIPS	TIPS	Cohen's d	No TIPS	TIPS	Cohen's d
	Mean (SD)			Mean (SD)		
Ascites	0.67 (0.47)	0.96 (0.21)	−0.636	0.69 (0.46)	0.88 (0.33)	−0.481
COPD	0.09 (0.28)	0.22 (0.42)	−0.437	0.09 (0.29)	0.12 (0.32)	−0.071
HCV	0.24 (0.43)	0.13 (0.34)	0.263	0.23 (0.42)	0.11 (0.32)	0.319
Portal vein thrombosis	0.13 (0.34)	0.09 (0.29)	0.138	0.13 (0.34)	0.11 (0.31)	0.080
HBV	0.25 (0.43)	0.39 (0.50)	−0.332	0.26 (0.44)	0.30 (0.46)	−0.097
HCC	0.55 (0.50)	0.35 (0.49)	0.398	0.53 (0.50)	0.36 (0.48)	0.359
Arterial hypertension	0.33 (0.47)	0.26 (0.45)	0.146	0.33 (0.47)	0.36 (0.48)	−0.069
Diabetes	0.40 (0.49)	0.48 (0.51)	−0.168	0.40 (0.49)	0.49 (0.50)	−0.180
Other etiologies	0.22 (0.41)	0.13 (0.34)	0.212	0.21 (0.41)	0.20 (0.40)	0.017
Previous abdominal surgery	0.16 (0.37)	0.09 (0.29)	0.198	0.15 (0.36)	0.12 (0.32)	0.099

Values are expressed as mean ± standard deviation (SD) for binary variables coded as 0/1; in this context, the mean represents the proportion of patients with the characteristic.

Cohen's d indicates the standardized mean difference between groups, with values closer to 0 representing better balance after IPTW. IPTW = inverse probability of treatment weighting; TIPS = transjugular intrahepatic porto-systemic shunt; COPD = chronic obstructive pulmonary disease; HCV = hepatitis C virus; HBV = hepatitis B virus; HCC = hepatocellular carcinoma.

### Postoperative Outcomes After Propensity Score Weighting

Post-transplant outcomes were compared between the TIPS and control groups in the IPTW-weighted pseudo-population (Table 4). After adjustment for baseline differences, no significant disparities were observed in total hospital stay (median [IQR]: 22 [16–23] vs. 21 [15–34] days; *P* = 0.246), ICU stay (6 [3–8] vs. 5 [3–10] days; *P* = 0.994), CCI (45.6 [22.6–49.1] vs. 34.6 [22.6–52.4]; *P* = 0.746), or in-hospital mortality (6.6% vs. 7.4%; *P* = 0.893). Notably, the rate of reoperation was significantly higher in the TIPS group after IPTW adjustment (17.4% vs. 8.0%, *P* = 0.003), a difference that was not apparent in the unweighted (univariate) analysis.

**Table 4:** Post-operative Outcomes in the IPTW-Weighted Pseudo-population.

Variable	TIPS	No TIPS	*P*-value
	Median [IQR] or %		
Total post-transplant hospital stay	22 [16–23]	21 [15–34]	ns
ICU stay after transplant	6 [3.0–8]	5 [3–10]	ns
CCI	45.6 [22.6–49.1]	34.6 [22.6–52.4]	ns
Reoperation	17.4%	8.0%	0.003
Intraoperative mortality during LT	3.7%	1.3%	ns
In-hospital mortality after LT	6.6%	7.4%	ns

Values are expressed as median [IQR] or weighted percentages derived from the IPTW-weighted pseudo-population.

IPTW = inverse probability of treatment weighting; TIPS = transjugular intrahepatic porto-systemic shunt; ICU = intensive care unit; CCI = comprehensive complication index; LT = liver transplantation; IQR = interquartile range. *P*-values refer to comparisons between groups (Mann–Whitney U test for continuous variables; chi-square or Fisher's exact test for categorical variables).

### Multivariable Logistic Regression Analysis

A parsimonious multivariable logistic regression model was constructed in the IPTW-weighted pseudo-population to identify predictors of in-hospital mortality (Table 5). The TIPS variable was forcibly retained in the model, along with two additional covariates selected through the LASSO procedure. Among these, previous abdominal surgery emerged as the only independent predictor of in-hospital mortality (OR: 3.83, 95% CI: 1.28–11.44, *P* = 0.016). Neither TIPS (OR: 0.91, 95% CI: 0.19–4.32, *P* = 0.902) nor HCC (OR: 0.77, 95% CI: 0.28–2.11, *P* = 0.607) were significantly associated with mortality. These results indicate that, after adjustment for baseline and intraoperative factors, pretransplant TIPS was not an independent determinant of early post-transplant death.

**Table 5:** Parsimonious Logistic Regression Model (IPTW-Weighted).

Variable	Beta	SE	OR	95% CI		*P*-value
				Lower	Upper	
Previous abdominalsurgery	1.342	0.559	3.826	1.279	11.440	0.016
HCC	−0.265	0.515	0.767	0.280	2.106	ns
TIPS	−0.098	0.797	0.906	0.190	4.322	ns

Beta = regression coefficient; SE = standard error; OR = odds ratio; 95% CI = 95% confidence interval; HCC = hepatocellular carcinoma; TIPS = transjugular intrahepatic porto-systemic shunt. Variables included in the model were previous abdominal surgery, hepatocellular carcinoma (HCC), and pre-transplant Transjugular Intrahepatic Porto-systemic Shunt (TIPS). *P*-values <0.05 were considered statistically significant.

## DISCUSSION

A major anticipated benefit of TIPS placement in patients listed for liver transplantation is the extension of the time window during which a graft can be allocated. This concept is indirectly supported by the meta-analysis by Larrue *et al.*, which demonstrated a reduction in further hepatic decompensation and improved two-year survival after TIPS.^23^ Consistently, retrospective analyses of the United Network for Organ Sharing (UNOS) registry showed that patients with TIPS had a lower likelihood of waitlist mortality and transplantation than the controls,^8^ and that those undergoing transplantation with a pre-existing TIPS had substantially longer waitlist times.^24^ Therefore, an important drawback of TIPS is the associated improvement in MELD score, which may paradoxically deprioritize patients with advanced portal hypertension in access to a graft.^8^^,^^24^

From the perspective of post–LT outcomes, the main finding of our study is that we failed to demonstrate a protective or detrimental effect of TIPS in our study population, and only previous abdominal surgery was predictive of in-hospital mortality. However, TIPS placement allows comparable outcomes to be achieved in a more complex patient population characterized by a higher burden of portal hypertension–related complications. Studies on the impact of pre-transplant TIPS on LT outcomes date back to the early 1990s^25^, ^26^, ^27^, ^28^, ^29^, ^30^, ^31^, ^32^, ^33^, ^34^, ^35^, ^36^, ^37^, ^38^, ^39^, ^40^, ^41^, ^42^, ^43^, ^44^, ^45^, ^46^ Most are retrospective, single-center studies, with sample sizes of TIPS recipients ranging from 7 to 130 patients, except for one registry-based study.^32^ Overall, the majority of studies found no clear benefit of pretransplant TIPS on operative time, transfusion requirements, hospital stay, postoperative complications, or graft survival. Notable exceptions include two studies reporting improved 1-year graft survival: the University of Wisconsin study (93% vs. 77% in 34 TIPS patients)^27^ and the Royal Free Hospital study in London (85.2% vs. 75.3% in 61 patients).^40^ However, survival in control groups was lower than typical, possibly exaggerating the apparent benefit. Large single-center studies from Marseille and Cleveland found no significant differences in perioperative outcomes or graft and patient survival,^42^^,^^45^ Matsushima *et al.* demonstrated increased hepatic venous flow after reperfusion in TIPS patients, but this did not translate into clinical benefits.^45^ A UNOS registry analysis of 1366 TIPS recipients reported similar 30-day mortality but longer hospital stays.^24^ A 2022 meta-analysis pooling 1885 patients from 13 studies confirmed no significant differences in most outcomes, with the only notable benefit being reduced fresh-frozen plasma use, largely driven by a small study of 8 patients.^47^ Many of the studies available in the literature refer to TIPS procedures performed using conventional stents. Overall, these data align with our findings.

A key confounding factor in previous studies is the selection of control groups, as TIPS recipients often present with more severe portal hypertension despite similar MELD scores. Most studies were single-center, which allows partial control of procedural variables but cannot fully account for all confounders. While Matsushima applied propensity score matching, others relied on multivariable Cox regression. In our study, we used an IPTW approach to balance covariates across a pseudo-population and confirmed the findings with multivariable logistic regression for in-hospital mortality, ensuring the most robust comparison possible.

Further information on this topic will be provided by the global IMPROVEMENT study, which included 5218 liver transplant candidates from Europe, Asia, Oceania, and the Americas. This study employed a combined retrospective and prospective design and performed a dedicated analysis of patients with TIPS.^48^

One strength of TIPS placement in LT candidates is its potential to prevent or facilitate the management of possible PVT.^6^ PVR-TIPS has emerged as a promising strategy in patients with complex portal vein anatomy, allowing restoration of portal flow and facilitating transplantation in otherwise challenging cases.^7^ However, this approach requires advanced interventional expertise and may be associated with a transient worsening of liver function. At our institution, PVR-TIPS is not routinely adopted and is reserved for highly selected cases.

A key challenge of TIPS in the transplant setting is stent misplacement or migration. Reported migration rates vary from 13% to 32% across studies.^40^^,^^42^^,^^45^ Migration can increase surgical complexity, potentially requiring a venous jump graft or modified anastomosis. Regarding outcomes, Guerrini and Matsushima reported reduced graft or patient survival with misplaced TIPS^40^^,^^45^ whereas Barbier found no significant impact. In our cohort, 30% of TIPS procedures had migrated at the time of transplantation, leading to increased surgical complexity, yet this did not translate into any negative impact on patient or graft outcomes.

One of the strengths of our study is the use of IPTW and a LASSO-based multivariable approach to address the limited sample size of the TIPS group. Another key strength is that patients undergoing TIPS received controlled-expansion stents, which offer an advantage over traditional stents by allowing underdilation, thereby reducing the incidence of hepatic encephalopathy without compromising clinical efficacy.^49^

The study is not without limitations. The single-center retrospective design may limit generalizability. In addition, the relatively small number of patients undergoing pretransplant TIPS may have limited the statistical power to detect differences in less frequent outcomes. Although IPTW and a LASSO-based multivariable approach were applied to mitigate confounding, residual unmeasured confounding cannot be completely excluded. Despite these limitations, the study provides contemporary, real-world evidence on the safety and perioperative impact of pretransplant TIPS.

In conclusion, although TIPS was misplaced in a minority of cases, this did not adversely affect outcomes. Preoperative TIPS allows patients with severe portal hypertension to achieve postoperative outcomes comparable to those of non-TIPS recipients, potentially expanding the pool of candidates who might otherwise be considered unsuitable for transplantation. However, TIPS does not appear to confer a benefit on the post-LT survival and should therefore be reserved for indications supported by current clinical guidelines. Only a randomized, multicenter, prospective trial could definitively clarify the impact of pre-transplant TIPS on LT outcomes.

## Credit authorship contribution statement

Conceptualization; Marco Biolato, Alfonso Avolio.

Data curation; Tiziano Galasso, Lucia Monastero, Antonio Liguori, Annamaria Amodeo.

Formal analysis; Quirino Lai; Roles/Writing - original draft; Marco Biolato

Supervision. Salvatore Agnes, Antonio Gasbarrini.

Writing - review & editing. Giuseppe Marrone, Luca Miele, Andrea Contegiacomo, Roberto Iezzi, Gabriele Spoletini, Antonio Grieco, Maurizio Pompili, Alfonso Avolio.

## Funding

None.

## Declaration of competing interest

The authors declare that they have no known competing financial interests or personal relationships that could have appeared to influence the work reported in this paper.
