# Supplementary material for: Liver Transplant Outcomes in Patients With Transjugular Intrahepatic Portosystemic Shunt: A Retrospective Cohort Study With Propensity Score Analysis
Source: J Clin Exp Hepatol. 2026 May 15;16(4):103561. doi: 10.1016/j.jceh.2026.103561 (PMC13266115; doi:10.1016/j.jceh.2026.103561)
Supplement: Supplementary file 1 [file mmc1.docx]

| **Impact of TIPS placement in patients awaiting transplant.** | | | |
| --- | --- | --- | --- |
| **Variable** | **Before TIPS** | **4 weeks after TIPS** | **p-value** |
| Bilirubin (mg/dl), median ± IQR | 2.0 ± 1.0 | 3.0 ± 2.0 | 0.009 |
| INR, median ± IQR | 1.0 ± 0.0 | 1.0 ± 1.0 | ns |
| Creatinin (mg/dl), median ± IQR | 1.0 ± 1.0 | 1.0 ± 1.0 | ns |
| Sodium (mEq/l), median ± IQR | 134.5 ± 8.0 | 134.0 ± 11.0 | ns |
| Albumin (g/dl), median ± IQR | 3.2 ± 0.6 | 2.6 ± 0.5 | ns |
| Hemoglobin (g/dl), median ± IQR | 9.0 ± 3.0 | 10.0 ± 1.0 | ns |
| Ammoniaemia (gamma/dl), median ± IQR | 75 ± 17 | 93 ± 94 | ns |
| Furosemide dose (mg/d), median ± IQR | 75 ± 72 | 37.5 ± 25 | ns |
| Antialdosteronic dose (mg/d), median ± IQR | 200 ± 188 | 100 ± 113 | 0.038 |
| MELD, median ± IQR | 11.5 ± 4.0 | 13.0 ± 4.0 | ns |
| MELDNa, median ± IQR | 16.5 ± 8.0 | 17.0 ± 8.0 | ns |
| Mann–Whitney U test.  Abbreviations: TIPS: Transjugular Intrahepatic Porto-systemic Shunt. MELD: Model for End-Stage Liver Disease; MELDNa: MELD-sodium. | | | |
